# Genomic Insights into an Environmental *Vibrio parahaemolyticus* Biofilm Isolate: Deciphering Alternative Resistance Mechanisms and Mobilizable Genetic Elements

**DOI:** 10.3390/antibiotics14101005

**Published:** 2025-10-10

**Authors:** Huiyu Liu, Yujian Dong, Zhongyang Lin, Olivier Habimana

**Affiliations:** 1 Biotechnology and Food Engineering Program, Guangdong Technion-Israel Institute of Technology, Shantou 515063, China; liu09756@gtiit.edu.cn (H.L.); dong08188@alumni.gtiit.edu.cn (Y.D.); 2 Department of Biology, College of Science, Shantou University, Shantou 515063, China; linzy@stu.edu.cn; 3 Faculty of Biotechnology and Food Engineering, Technion-Israel Institute of Technology, Haifa 3200003, Israel

**Keywords:** *Vibrio parahaemolyticus*, antibiotic resistance, biofilm, mobile genetic elements, one health, transposons, efflux pumps, environmental reservoir, comparative genomics

## Abstract

Background/Objectives: Biofilms are key in spreading antibiotic resistance in various ecosystems. This study employed comparative genomics to examine the resistance and adaptability mechanisms of the *Vibrio parahaemolyticus* strain Vaw-5, isolated from a seafood market biofilm. Methods: A comparative examination of Vaw-5 and 32 publicly available *V. parahaemolyticus* genomes identified a distinct set of genetic resistance characteristics. Results: Unlike clinical strains, Vaw-5 lacks acquired antimicrobial resistance genes like the *blaCARB* and *qnr* variations. Instead, its resistance potential is based on chromosomal alterations, efflux pump systems (*vmeAB*, *vcmD*), and a unique repertoire of 16 strain-specific transposons, including *Tn5501* and *Tn5393*, which are well-known vectors for antibiotic resistance gene (ARG) mobilization. Although not multidrug-resistant, Vaw-5 possesses unique genomic islands that share negligible homology with those of clinical strains, enriched with gene clusters for environmental adaptation, such as exopolysaccharide production and a fully functional Type VI Secretion System. Vaw-5 carries a distinctive plasmid with the resistance gene *aac(2′)-Ia*. Conclusions: Biofilm adaptation promotes structural integrity, inherent processes, and resistance above standard ARG acquisition. This study focuses on how biofilm communities in the food chain can operate as covert incubators for mobilizable resistance determinants, emphasizing the significance of ecological monitoring within a One Health paradigm to reduce possible public health hazards.

## 1. Introduction

The consumption of raw or insufficiently processed seafood constitutes a significant and persistent global public health dilemma, chiefly ascribed to pathogens elicited by the halotolerant microorganism *Vibrio parahaemolyticus* [1]. Vibriosis cases have been linked with heightened seawater thermal parameters, environmental alterations, and the ingestion of raw or insufficiently processed seafood, which amplifies exposure vulnerability [2,3]. Because of its strong ability to thrive and reproduce across many salinity levels, *V. parahaemolyticus* has become a common and important part of various marine ecosystems, posing a notable risk to human health and safety [4]. The pressing necessity for more stringent and comprehensive food safety rules within the seafood industry is underscored by persistent outbreaks linked to specific seafood sources [2]. This pathogen causes diverse clinical symptoms, from mild gastroenteritis to severe septicemia, underscoring its ability for swift spread via complex genomic mechanisms that are not fully understood [5].

*V. parahaemolyticus’* ability to synthesize biofilms—complex microbial aggregates held together by a self-produced extracellular matrix—demonstrates its sophisticated mechanisms and adaptability in various environments [6]. Microorganisms are protected by biolayers from various strains and host immune reactions [7,8]. This resilience significantly facilitates chronic infections and disease dissemination by allowing prolonged colonization of both biotic surfaces, like the human digestive tract, and abiotic surfaces, such as aquaculture equipment and food processing infrastructure [7]. Consequently, understanding the virulence of this organism and recognizing prospective therapeutic targets relies on clarifying the genetic mechanisms governing biofilm development.

Enhancements in next-generation sequencing and extensive analytical methodologies have significantly amplified the examination of bacterial genomic diversity and phylogenetic connections [9]. Comparative genomics has consequently emerged as a significant instrument, facilitating the concurrent examination of myriad genomes for an extensive comprehension of the mechanisms of evolution influencing bacterial populations [10]. To comprehend microbial dissemination of characteristics and evolution, one must scrutinize the interaction of vertical inheritance and horizontal gene transfer (HGT).

Although the number of sequenced *V. parahaemolyticus* genomes is increasing, few genome-based comparative studies investigating the roles of HGT and vertical inheritance from different ecological sources, especially within biofilms, have been performed [11]. Additional inquiries are required to bridge this divide and discern the genetic elements that contribute to this microorganism’s virulence and adaptability [12]. This study addresses the gap by focusing on strain Vaw-5, a distinct isolate of *V. parahaemolyticus* identified inside a biofilm community in a holding tank of Japanese mantis shrimp (*Oratosquilla oratoria*) at a seafood market in Shantou, China. Vaw-5, identified as an uncharacterized strain through genomic analysis, presents a significant opportunity to clarify the genetic framework that enables biofilm niche adaptability and to examine the contributions of vertical inheritance and horizontal gene transfer to its evolutionary history [13].

Preliminary results suggest that the genetic makeup of Vaw-5 differs from recorded strains of *V. parahaemolyticus* [13], and requires further examination. We propose that strain Vaw-5′s unique adaptability to its biofilm environment arises from both vertical inheritance, which maintains essential genomic functions, and significant horizontal gene transfer, facilitating the acquisition of a diversified accessory genome. Regarding its accessory genome composition, including phage-associated genes, mobile genetic elements (MGEs), antibiotic resistance genes (ARGs), and the structure of genomic islands, we anticipate that this interaction has produced a unique genomic signature that differentiates it from other isolates The primary objective of this research is to perform an extensive genomic analysis of the *V. parahaemolyticus* strain Vaw-5 and various isolates from clinical, environmental, and animal origins. To accomplish this, the pangenome must be mapped, the phylogenetic connections need to be determined, and the supplementary genome meticulously examined, concentrating on phage-related genes, genomic islands, mobile genetic elements, and antibiotic resistance (AR) genes. This investigation aspires to clarify the unique genomic characteristics of Vaw-5, focusing on the hereditary foundation for resistance strategies and their activation. Our results present novel viewpoints on the genomic underpinnings of biofilm acclimatization within an ecological context and its prospective ramifications for the endurance and dissemination of resistance factors.

## 2. Results

Focusing on resistance strategies, our comparative genomic examination of 33 complete *V. parahaemolyticus* genomes—including the biofilm-derived strain Vaw-5—yielded essential revelations regarding the genetic variability, evolutionary history, and niche-specific modifications of the species.

### 2.1. An Open Pangenome Underpins Extensive Genetic Diversity

**Figure 1:**
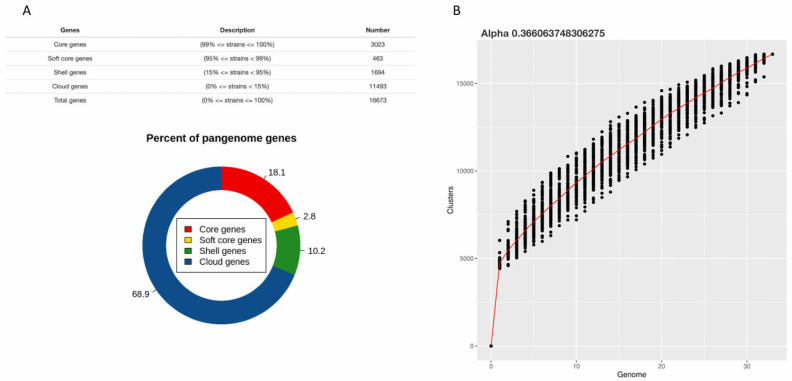
*Vibrio parahaemolyticus* pangenome composition and growth. (**A**) Pie chart depicting the distribution of genes across core (present in ≥99% of strains), soft-core (95–99%), shell (15–95%), and cloud (<15%) genome categories for 33 strains, reflecting the extensive genomic plasticity within the species. (**B**) Heaps’ law model of pangenome growth. The fitted model (n = k · N^α) with an exponent α = 0.366 confirms a highly open pangenome, indicating that the total gene pool is far from saturated and sequencing additional genomes will continue to discover a high number of new genes.

A complete examination of the 33 genomes revealed a substantial, open pangenome with 16,673 genes. Mathematical modeling using Heaps’ rule showed a highly open structure (α = 0.366) that is expected to develop with additional sequencing (Figure 1). The core genome, encompassing 3023 alleles (18.1%), was discernible in ≥99% of variants. Of them, 92% encoded essential metabolic processes. On the other hand, the cloud category dominated the auxiliary genome, accounting for 11,493 genes (68.9%) with strain-specific distribution patterns. Statistical examination of the accessory genome unveiled that the allocation of mobile genetic elements was not homogeneous. Genomic islands were significantly enriched in the cloud genome (*p* = 4.29 × 10^−5^), indicating that strain-specific gene content is heavily influenced by the acquisition of large, horizontally acquired DNA segments. In contrast, transposons were not significantly enriched and were distributed nearly randomly across the pangenome (*p* = 0.937). Vaw-5 contributed 84 unique genes to the pangenome.

### 2.2. Phylogenetic Analyses Reveal Niche-Driven Evolution

**Figure 2:**
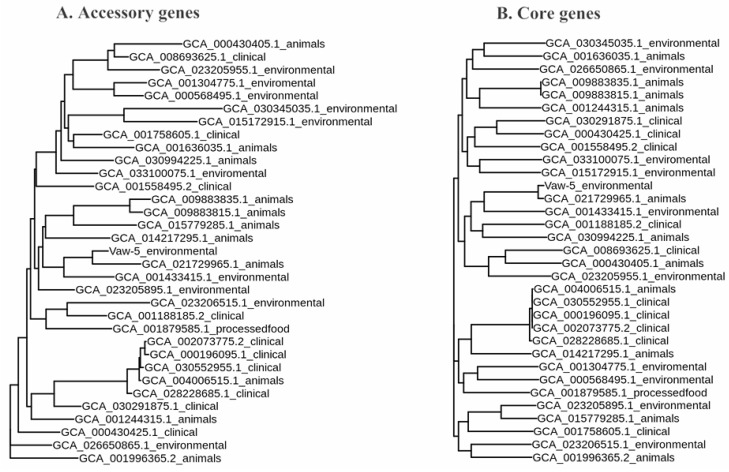
Maximum-likelihood phylogenetic trees of *Vibrio parahaemolyticus* strains based on (**A**) accessory and (**B**) core genomes. The accessory genome phylogeny shows strong clustering by ecological niche (environmental, clinical, animal), while the core genome phylogeny reveals deeper evolutionary relationships with less resolution for recent ecological divergence.

We constructed trees utilizing both accessory and core genomic data to better understand the evolutionary dynamics and phylogenetic linkages within *V. parahaemolyticus*. The additional genomic phylogenetic examination revealed a significant, niche-specific congregation, with ecological, clinical, and zoological isolates constituting discrete clades (Figure 2A). In contrast, the core genome phylogeny uncovered a subtler grouping by isolation origin, with strains originating from medical, ecological, and zoological settings interspersed throughout the phylogenetic dendrogram (Figure 2B).

### 2.3. Strain Vaw-5 Exhibits a Unique Genomic Profile

**Figure 3:**
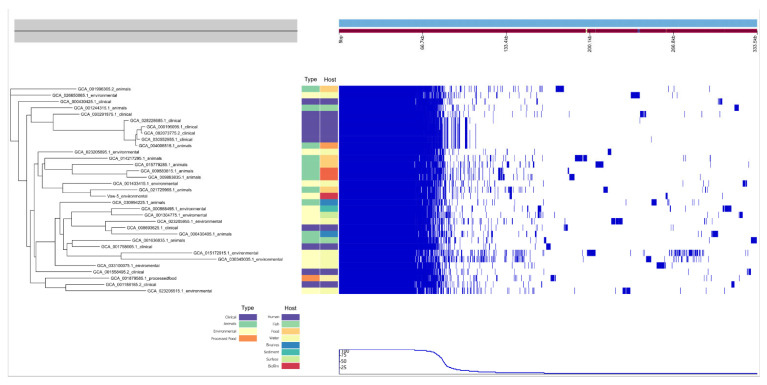
Phylogenetic tree of *Vibrio parahaemolyticus* strains based on core genome alignment, with adjacent accessory genome matrix. Strains are annotated by isolation type (clinical, environmental, animal, processed food) and host source. The genome matrix illustrates gene presence (blue) or absence (white) across accessory genomic regions, highlighting niche-specific adaptations.

Multilocus sequence typing (MLST) analysis assigned Vaw-5 to sequence type (ST) 424, a type shared with an animal isolate (GCA_021729965.1), suggesting a common phylogenetic ancestry (Table 1). Nevertheless, accessory genomic examination disclosed considerable variation between Vaw-5 and all other strains, encompassing its ST equivalent (Figure 3). This distinct accessory gene profile, lacking a direct link between strain origin and gene composition, indicates significant adaptation to its particular biofilm environment, primarily influenced by horizontal gene transfer (HGT). To further investigate this, we expanded our core genome analysis to include four additional housekeeping genes with diverse functions (*yeiP*, *recO*, *brkB*, *ompH*). The resulting phylogenies showed varying degrees of congruence with isolation source, from strong vertical inheritance patterns in *yeiP* to patterns indicative of extensive HGT in the virulence-associated *brkB* gene (Supplementary Figures S1–S4). The phylogenetic position of Vaw-5 across these trees is consistent with a complex evolutionary history shaped by both vertical inheritance and HGT, facilitating its unique adaptation.

### 2.4. A Distinct Antibiotic Resistance Gene Profile Highlights Alternative Strategies

**Figure 4:**
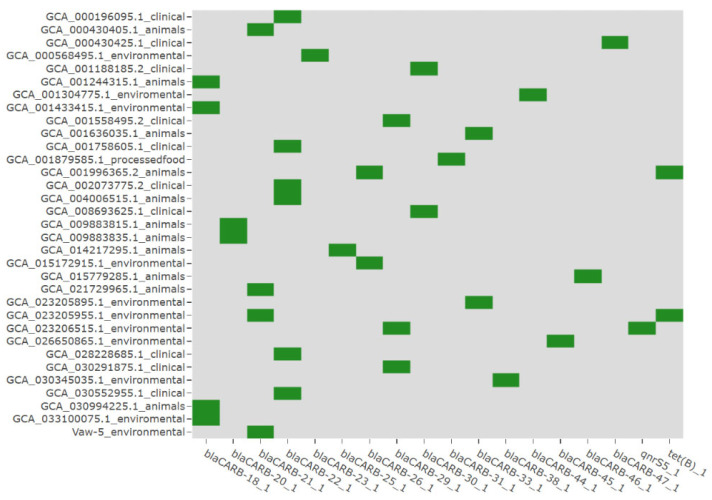
Comparative analysis of antibiotic resistance genes (ARGs) across *Vibrio parahaemolyticus* strains. The heatmap illustrates the presence (green) or absence (gray) of key ARGs, highlighting the distinct resistance profiles between clinical isolates, which harbor acquired genes, and the environmental isolate Vaw-5, which relies on alternative mechanisms.

Our research revealed significant variance in antibiotic resistance genes (ARGs) between strains, particularly between Vaw-5 and clinical isolates (Figure 4). Clinical strains had significantly more absorbed resistance genes such as *blaCARB*, *tet(B)*, and *qnrS5*, indicating direct antibiotic selection pressures. In contrast, Vaw-5 included genetic sequences that encoded many resistance pathways but lacked these clinically relevant resistance determinants. Genomic investigation revealed chromosomally encoded efflux pump systems (*vmeAB*, *vcmD*) and a stress response regulator (*rpoS*), while failing to identify frequently acquired ARGs such as blaCARB and qnr variants observed in clinical isolates.

### 2.5. Mobile Genetic Elements and Genomic Islands Suggest Potential for Antimicrobial Resistance Mobilization

**Figure 5:**
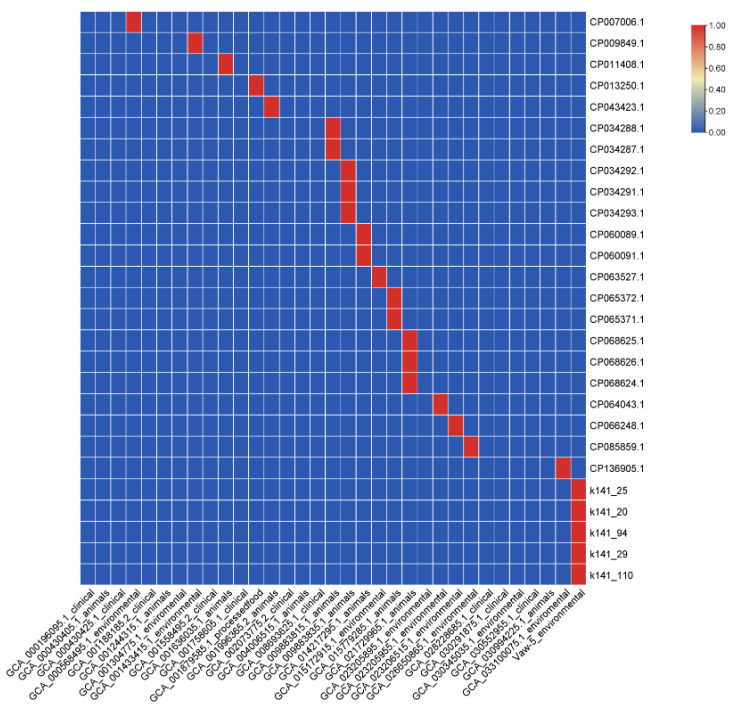
Plasmid distribution across *Vibrio parahaemolyticus* strains. The heatmap compares plasmid similarity blocks, with unique regions in Vaw-5 (highlighted), suggesting horizontal gene transfer events and niche-specific genetic acquisitions.

**Figure 6:**
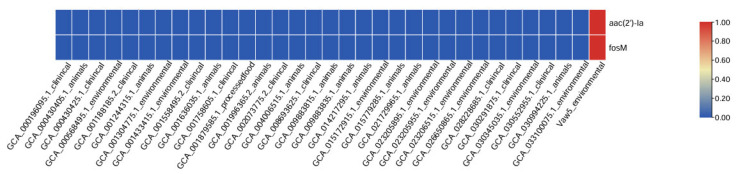
Plasmid similarity and unique gene content across *Vibrio parahaemolyticus* strains. The heatmap highlights the distinct plasmid profile of the biofilm isolate Vaw-5, including a plasmid carrying the *aac(2′)-Ia* resistance gene, indicating niche-specific acquisition and retention of mobile genetic elements.

Analysis of MGEs indicates their key function in Vaw-5 genomic flexibility. Plasmid comparison showed that Vaw-5 contained a distinct set of plasmids compared to clinical and environmental isolates (Figure 5 and Figure 6). Notably, Vaw-5 lacked several plasmids common in clinical isolates that often carry well-characterized virulence and resistance determinants (e.g., *blaCARB*, *qnrVC*). However, it possessed unique plasmid content, including one carrying the aminoglycoside resistance gene *aac(2′)-Ia*, suggesting a niche-specific process of MGE acquisition and retention.

In parallel, chromosomal analysis revealed that Vaw-5 possesses unique genomic islands that share negligible sequence homology with those of clinical strains. These islands were enriched with gene clusters involved in environmental adaptation, including those for lipopolysaccharide (LPS) and O-antigen biosynthesis (e.g., *neuB*, *neuC*, *waaA*, *rfbABCD*), a complete Type VI Secretion System (T6SS), and various efflux pumps (Supplementary Table S1). The unique architectural framework and genetic composition of these islands provide clear proof for lateral gene transfer occurrences that have influenced the accessory genome of Vaw-5.

**Figure 7:**
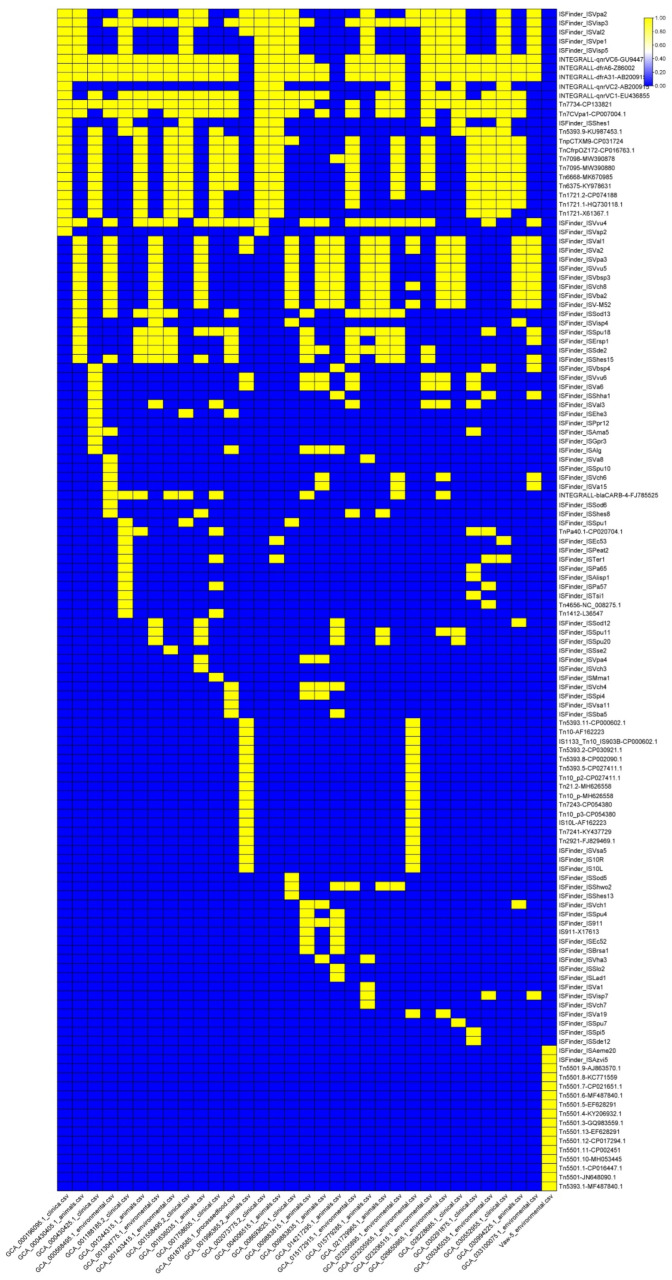
Distribution of insertion sequences (ISs) and transposons across *Vibrio parahaemolyticus* genomes. The heatmap shows the presence/absence of mobile genetic elements, with Vaw-5 harboring 16 unique transposons (highlighted in red), underscoring the role of MGEs in genomic plasticity and environmental adaptation.

**Figure 8:**
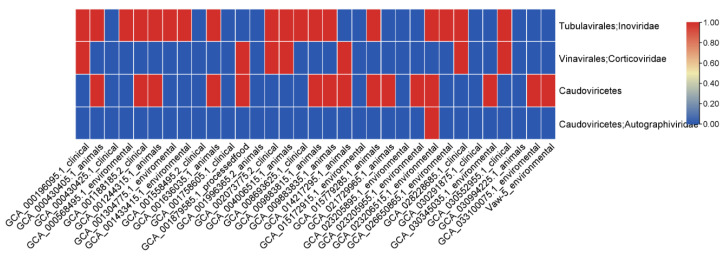
Diversity of phage-related genes across *Vibrio parahaemolyticus* strains. The heatmap depicts strain-specific variations, with Vaw-5 displaying a distinct profile indicative of specialized phage interactions within its biofilm niche.

Most notably, a comprehensive analysis of insertion sequences (ISs) and transposons revealed that Vaw-5 possesses a highly distinct profile characterized by 16 strain-specific transposons (Figure 7). Among these were Tn5501 and Tn5393, which are thoroughly recorded for their function in seizing and transferring resistance genes. This unique transposon repertoire, co-occurring with a distinctive configuration of phage-associated genes (Figure 8), underscores a genomic architecture in Vaw-5 with high inherent plasticity and a latent potential for the mobilization of adaptive traits, including antimicrobial resistance.

## 3. Discussion

*V. parahaemolyticus* is a notable and prevalent bacterium that is responsible for gastroenteritis associated with seafood on a global scale, commonly associated with the consumption of raw or inadequately cooked seafood that poses health risks [2,3]. The ability to create biofilms on seafood processing surfaces and aquaculture equipment is a risk factor for the continued contamination of an establishment or outbreaks [6,7]. Investigation of biofilm adaptations in environmental isolates such as Vaw-5 is, therefore, essential for a better understanding of how this pathogen is maintained in the food chain and escapes from control.

This comprehensive genome study provides previously unknown insights into the adaptation strategies of *V. parahaemolyticus* strain Vaw-5, which was isolated from a complex biofilm environment in seafood market infrastructure. Our comprehensive study elucidates the importance of horizontal gene transfer (HGT) and hereditary transmission in ecological niche-specific modifications, which possesses considerable ramifications for biofilm proliferation and resilience. A comprehensive description of these findings is presented below, accentuating Vaw-5′s distinctive genomic architecture, contrasting its characteristics with those of clinical isolates, and highlighting substantial informational voids that necessitate further inquiry.

### 3.1. Evolutionary Mechanisms and Pangenome Dynamics

*V. parahaemolyticus* populations have an open pangenome structure (α = 0.366), similar to adaptive radiation patterns found in marine vibrios [14]. This genomic structure facilitates swift adaptation through horizontal gene transfer. Strain Vaw-5 exemplifies this evolutionary strategy, as it has 84 unique genes that set it apart from clinical and environmental comparators; many of these genes are expected to perform roles in ecological adaptation, emphasizing the relevance of the accessory genome in supplying the genetic variety required for niche specialization. The projected activities of twelve of these distinct genetic components in biofilm development and maintenance indicate distinctive adaptations to seafood market conditions. Azeem et al. (2025) assert that biofilm-related genes facilitate surface adhesion, enhance nutrient absorption amidst variable conditions, and provide protection against environmental hazards in food processing environments [7]. The presence of mobile genetic elements in accessory genome components suggests a mechanism for maintaining and spreading genetic diversity in bacterial populations (*p* < 0.001). This pattern of genetic flexibility indicates a fundamental evolutionary strategy in *V. parahaemolyticus*, allowing for rapid environmental adaptation while keeping essential physiological activities [15]. The existence of substantial extragenomic fragments in this ecologically proficient organism exemplifies the evolutionary correlation of genetic conservation with adaptive progression.

### 3.2. Understanding Adaptation Pathways Using Phylogenetic Analysis

Our dual phylogenetic method revealed different evolutionary paths for accessory and core genomes. The accessory genome phylogeny showed significant niche-specific grouping (Figure 2A), indicating that HGT is a directed process affected by environmental factors rather than random acquisition. This phenomenon indicates that the procurement of novel genes is not a fortuitous event, but instead a systematic procedure regulated by selective influences distinctive to a specific ecosystem. Importantly, ecological isolates such as Vaw-5 formed a distinct clade that differed from clinical and animal forms, emphasizing the function of horizontal gene transfer (HGT) in ecology. This is congruent with the findings of Tokuda and Shintani (2024), who discovered that habitat-specific selection is typically represented in MGE-mediated gene flow [14]. In contrast, in accordance with the preserved characteristics of these genes, the fundamental genomic phylogeny (Figure 2B) elucidated more profound evolutionary connections but constrained clarity regarding recent ecological divergence. The contrasts between these phylogenies demonstrate how the core and supplemental genomes interact to reconstruct an organism’s evolutionary history. This dichotomy is best exemplified by Vaw-5′s position in both trees: its core genome preserves ancestral lineage signals that are ubiquitous across environments, yet its accessory genome clusters with isolates connected to biofilms.

### 3.3. Distinct Evolutionary Approaches: Clinical Isolates Versus Biofilm Isolate

The genetic variations between clinical isolates and Vaw-5 underscore distinct adaptation processes shaped by their respective environmental pressures. The existence of resistance genes like *blaCARB* and *qnrVC* on plasmids in clinical strains indicates significant antibiotic selection pressures in healthcare environments [16]. The genomic profile of Vaw-5 indicates a unique resistance mechanism adapted for biofilm settings. This strategy is characterized not by the acquisition of classic, high-level resistance plasmids, but by a combination of intrinsic mechanisms and a unique mobilizable genetic arsenal. The presence of chromosomal efflux pumps (*vmeAB*, *vcmD*), stress response regulators (*rpoS*), and the physical barrier of the biofilm itself likely reduces the selective pressure to maintain specific, acquired ARGs commonly seen in clinical strains [17].

This evolutionary divergence is further evidenced by Vaw-5′s distinct MGE profile. While it lacks typical clinical resistance plasmids, Vaw-5 has acquired a unique plasmid carrying the *aac(2′)-Ia* resistance gene, indicating a niche-specific pathway of MGE uptake. More broadly, its chromosomal architecture has been significantly shaped by HGT, as demonstrated by unique genomic islands sharing less than 30% homology with clinical strains. These islands are enriched with gene clusters for environmental persistence, including those for exopolysaccharide and lipopolysaccharide biosynthesis (e.g., *neuB*, *neuC*, *rfbABCD*)—critical for biofilm matrix formation and immune evasion—and a complete Type VI Secretion System (T6SS) for microbial competition. Crucially, Vaw-5′s genome exhibits high inherent plasticity, underscored by its unique repertoire of 16 strain-specific transposons, including *Tn5501* and *Tn5393*. These elements are well-documented vectors for gene capture and dissemination [18], and their presence, alongside a distinct phage-related gene profile, signifies a genomic architecture with a high latent potential for the mobilization of adaptive traits.

Consequently, while clinical strains evolve under direct antibiotic pressure, often accruing resistance genes on conserved plasmids, Vaw-5 exemplifies an environmental adaptation strategy. This strategy prioritizes biofilm-associated structural integrity and competitive prowess, supported by a genetically fluid accessory genome. This plasticity equips Vaw-5 not with a fixed set of high-level resistance genes, but with a versatile toolkit from which resistance determinants can be mobilized or acquired as needed, positioning it as a potential “stealth incubator” for resistance factors in the environment.

### 3.4. Implications of the One Health Concept and Antimicrobial Resistance

Together, the Vaw-5 findings provide compelling evidence for the presence of complicated alternate resistance mechanisms that go beyond the established hypothesis of acquired ARGs. This complex strategy combines a genetic arsenal predisposed to mobilization with the intrinsic resistance afforded by the biofilm structure. While the distinct catalog of MGEs, particularly the *Tn5501* and *Tn5393* transposons, is a potential source of resistance propagation, the presence of functioning efflux systems provides a route for antimicrobial extrusion. The *aac(2′)-Ia* resistance allele is intentionally situated within its genomic island, in proximity to integrase alleles, underscoring its mobilizability. This is paramount in accordance with the One Health paradigm, which recognizes the interrelation of the ecological, animal, and human health systems. Vaw-5 was isolated from the seafood market environment, a critical interface where bacteria from humans, water, aquatic animals, and processing surfaces interact. Although not multidrug resistant in and of itself, a biofilm-adapted strain like Vaw-5 can act as a “stealth incubator” for resistance factors in such a scenario. It may bridge the gap between human pathogens and the environmental resistome by exploiting horizontal gene transfer (HGT) to transfer its mobilizable genetic payload to more pathogenic or clinically relevant bacteria in the same environment. The fact that *V. parahaemolyticus* is frequently associated with outbreaks and seafood items, showing that it moves up the food chain, heightens this risk [19,20,21]. As a result, environmental isolates like as Vaw-5 are a major issue due to their genetic potential for resistance gene mobilization and physical biofilm barriers that inhibit antibiotic penetration. These environmental stresses can thus serve as reservoirs of shareable resistance attributes. Through horizontal gene transfer in settings like seafood markets or aquaculture, these determinants could be acquired by human-pathogenic strains, potentially leading to the emergence of resistant clinical pathogens that complicate treatment. Our data show the need to expand antimicrobial resistance surveillance to include environmental and biofilm-derived populations, as they are a vital reservoir in the One Health continuum.

### 3.5. Key Knowledge Gaps and Prospects

Future studies must address a variety of constraints, despite the fact that our genome analysis provides compelling ideas about Vaw-5′s biofilm adaptations. To confirm the functional significance of revealed genomic characteristics, future work should include phenotypic antibiotic susceptibility profiling and comparative transcriptome analysis of sessile versus planktonic cells to directly link genetic potential to the biofilm lifestyle and its associated resistance. Moreover, quantitative biofilm formation inquiries under various ecological circumstances, gene deletion evaluations directed at potential biofilm-related genes, and transcriptomic analyses of different developmental phases are essential [22]. Furthermore, the singular-isolate framework—concentrating exclusively on Vaw-5—restricts the generalizability of our results to the wider heterogeneity of biofilm-associated *V. parahaemolyticus* populations. Future inquiries should include various biofilm strains from distinct ecological environments to embody a more representative genomic diversity.

### 3.6. Broader Implications

Our findings have major implications for other domains. The Vaw-5 genome provides a model for understanding how bacteria adapt to surface-associated lifestyles in microbial ecology, leading to general parameters for biofilm adaptation [6]. Genetic variations between clinical and environmental isolates emphasize the importance of focused, niche-specific intervention techniques for public health protection [2]. Vaw-5′s presence in seafood market infrastructure emphasizes the critical need for biofilm control in food processing environments for aquaculture and food safety by reducing the possibility of pathogen persistence and resistance gene transfer [13].

## 4. Materials and Methods

### 4.1. Biofilm Sample Collection and Bacterial Isolation

The *Vibrio parahaemolyticus* strain Vaw-5 was isolated from a biofilm on the surface of a Japanese mantis shrimp (*Oratosquilla oratoria*) holding tank in a wet market in Shantou, China, as previously described [13]. Biofilm was sampled by aseptically scrubbing a defined area of 50 cm^2^ of the tank surface with a sterile cellulose sponge swab (Genstone Biotech, Beijing, China) that had been hydrated in 10 mL of sterile saline solution (0.85% NaCl). The sponge was carefully placed in a sterile 50 mL centrifuge tube to preserve its integrity and transported to the laboratory within an hour to meet testing protocols. For isolation, the sponge in the centrifuge tube was homogenized by vortexing for 30 s, and the homogenate was serially diluted in saline and plated on TCBS agar (Huankai Microbial, Guangzhou, China). A negative control, consisting of a sterile sponge processed identically, was included during homogenization and plating to confirm the absence of environmental contamination. Cultures were incubated at 37 °C for a duration of 18 to 24 h. Characteristic green colonies were isolated directly from TCBS agar to capture a representative isolate from the biofilm community. A single purified isolate was then enriched in alkaline peptone water (APW; pH 8.6, 20 g each of peptone and NaCl per liter), followed by preservation in 1:1 mixture of enriched culture and 50% glycerol APW stock at −86 °C, for a glycerol stock preparation. A single purified isolate was selected for whole-genome sequencing.

### 4.2. Genomic DNA Extraction and Quality Control

Genomic DNA (gDNA) was extracted from a fresh overnight culture of Vaw-5 grown in APW at 37 °C with shaking at 200 rpm. The genomic DNA extraction was conducted utilizing the PureLink™ Genomic DNA Mini Kit (Invitrogen, Waltham, MA, USA), in strict adherence to the manufacturer’s directives applicable to Gram-negative bacteria. The integrity of the DNA was evaluated through spectrophotometric analysis utilizing a NanoDrop One (Thermo Fisher Scientific, Waltham, MA, USA), which produced A260/280 and A260/230 ratios of 1.92 and 2.15, correspondingly. The integrity of the isolated DNA was validated through electrophoresis conducted on a 0.8% agarose gel, revealing a distinct band of elevated molecular weight with negligible diffusion. A total of 1 μg of this superior-grade gDNA was submitted to Novogene Co., Ltd. (Tianjin, China) for library preparation and sequencing.

### 4.3. Library Preparation, Sequencing, and Primary Data Processing

Library preparation and sequencing provisions were conducted by Novogene. The qualified gDNA sample was randomly fragmented by sonication using a Covaris M220 to a target size of 350 bp. A sequencing library was constructed from the fragmented DNA with a process including end repair, A-tailing, adapter ligation, and PCR amplification. The final library was quality-controlled using an Agilent 5400 Fragment Analyzer System (Agilent Technologies, Santa Clara, CA, USA) and quantified by qPCR. The library was sequenced on an Illumina NovaSeq 6000 platform (Illumina, San Diego, CA, USA) utilizing an S4 flow cell and a 2 × 150 bp paired-end arrangement. Novogene’s standard bioinformatics pipeline was used for primary data analysis, including base calling and demultiplexing, which produced the raw FASTQ files for downstream analysis.

### 4.4. Genome Assembly, Quality Assessment, and Contamination Screening

The raw sequencing reads were subjected to primary quality control and adapter trimming by Novogene using fastp V.0.23.1 [23] to discard a paired read if (1) either one read was contaminated by an adapter; (2) more than 10% bases in either one read are uncertain; (3) 50% bases with Phred quality score less than 5 in either one read; achieving Q30 percentage of 94.39%. The subsequent reads were further quality-filtered using PRINSEQ [24] in TORMES V.1.3.0 [25] with --min_len 125 and default minimum average Phred score threshold. This procedure eliminated reads that were shorter than 125 base pairs. The quality-filtered reads were then assembled de novo using SPAdes V.3.15.4 [26] with the isolate flag enabled to reduce running time and improve assembly quality. Potential contamination was assessed by querying all assembled contigs against the NT database using Kraken2 V.2.1.2 [27] with a confidence threshold of 0.1; this analysis detected no significant non-Vibrio contamination. The resulting assembly was evaluated using QUAST V.5.2.0 [28]. Finally, assembly quality and completeness were assessed using CheckM V.1.2.2 [29] with the lineage_wf command and BUSCO V.5.4.3 [30] with the proteobacteria_odb10 dataset.

The final assembly for Vaw-5 had a total size of 5.17 Mbp distributed across 103 contigs. The assembly statistics included an N50 of 480,379 bp, an L50 of 4, and a largest contig of 1,192,045 bp. CheckM analysis indicated 99.8% completeness and 0.45% contamination. BUSCO analysis of the proteobacteria_odb10 dataset (*n* = 395) revealed a completeness of 99.8% (C: 99.8% [S: 99.6%, D: 0.2%], F: 0.0%, M: 0.2%). The assembly contained several large contigs corresponding to chromosomes I and II, alongside 5 smaller contigs predicted by geNomad [31] to be circular plasmids of 62 kb, 26 kb, 23 kb, 11 kb, and 10 kb. It is important to note that, as this assembly is based on short-read technology, these are considered chromosome-level scaffolds and draft plasmid sequences; their circularization was inferred from coverage depth and terminal repeat sequences but was not experimentally validated with long reads. The complete genome sequence was deposited under NCBI BioProject PRJNA1112528.

### 4.5. Comparative Genomic Dataset Curation

A representative comparative dataset of 32 complete *V. parahaemolyticus* genomes was downloaded from the NCBI RefSeq database on 27 November 2024 (Table 2). The criteria for genome selection comprised: (1) designation as ‘Complete Genome’ or ‘Chromosome’, (2) high-quality annotation availability, (3) diverse geographical representations, and (4) inclusion of primary isolation sources like clinical, environmental, and animal contexts. A comprehensive collection includes all thirty-two strains used in this study, with their NCBI Assembly IDs (GCA), BioSample IDs, and SRA accession numbers listed in Supplementary Table S1 for reference. All subsequent genomic analyses were performed on this combined dataset of 33 genomes, which includes the 32 public genomes and the novel Vaw-5 genome.

### 4.6. Genome Annotation and Gene Calling

To ensure consistency across the dataset, all 33 genomes were uniformly re-annotated using the TORMES V.1.3.0 pipeline [25]. The pipeline executed several key steps with specific parameters. Multi-Locus Sequence Typing (MLST) was conducted employing the mlst V.2.19.0 software (Seemann T, mlst, GitHub https://github.com/tseemann/mlst) (accessed on 31 July 2025).alongside the specific *V. parahaemolyticus* scheme. Antibiotic Resistance Genes (ARGs) were discerned utilizing ABRicate V.1.0.1 (Seemann T, Abricate, GitHub https://github.com/tseemann/abricate) (accessed on 3 January 2025) in conjunction with the ResFinder database V.4.7.2 [32] with minimum criteria of 80% nucleotide identity and 80% coverage. Similarly, Virulence Factors (VFs) were identified using ABRicate against the core DNA dataset of the Virulence Factor Database (VFDB) [33] using the same thresholds of 80% for both identity and coverage.

### 4.7. Pangenome and Phylogenetic Analysis of Genomes and Housekeeping Genes

Gene prediction of the genomes was executed using Prodigal V.2.6.3 (Doug Hyatt, https://github.com/hyattpd/Prodigal) (accessed on 3 January 2025). For pangenome and phylogenetic analysis of genomes, quick annotation was performed using Prokka V.1.14.6 [34] and Prokka bacteria and hmm databases with default parameters, including a minimum criteria of 80% peptide identity, and e-value threshold of 1 × 10^−9^. The subsequent GFF documents were analyzed using Roary V.3.13.0 [35], with a minimum BLASTp identity criterion of 95% (-i 95). Core gene definitions were set as follows: core (99% of strains), soft-core (95–99%), shell (15–95%), and cloud (<15%). The pangenome growth model was fitted to Heaps’ law (n = k · N^α) using a custom R script, which has been deposited in the Supplementary Materials; this script iteratively calculated the number of new genes added by each randomly permuted genome. For phylogenetic examination, the alignment of the fundamental genome (3023 genes) and the gene presence/absence matrix of the accessory genome produced by Roary were utilized to formulate a maximum likelihood phylogenetic tree and calculate support value by local bootstrap with FastTree [36]. Summary figures are generated by roary2svg (T.Seemann, https://github.com/sanger-pathogens/Roary/blob/master/contrib/roary2svg/roary2svg.pl) (accessed on 31 July 2025), and Phandango web tool [37] (accessed on 3 January 2025). Similarly, for housekeeping gene phylogenetic analysis, after gene prediction, the genome annotation was performed using Prokka [34] with default settings, which was supplied with a custom protein database containing 5,689,969 *Vibrio* RefSeq protein sequences downloaded from NCBI on 24 December 2024, using the --proteins option. Pangenome Explorer web tool [38] using Roary [35] assessed the gbk files at a 90% minimum BLASTp identity threshold (accessed on 29 July 2025).

### 4.8. Identification of Mobile Genetic Elements (MGEs) and Genomic Islands

The methodology utilized for the identification of Mobile Genetic Elements (MGEs) and Genomic Islands (GIs) was meticulously executed through the application of an extensive list of sophisticated bioinformatics programs specifically designed for these types of analyses. Plasmids and proviruses were identified and annotated using geNomad V.1.8.1 with the geNomadDB V.1.7 database [31] under default parameters. Insertion sequences (ISs) and composite transposons were identified using the TnCentral web tool [18] (accessed on 12 January 2025), which integrates the ISfinder [39] and Integrall [40] databases; hits were filtered for a minimum of 75% identity, and a loose e-value threshold of 10 of the reference transposon, and “Low complexity” and “Mask for lookup table only” filters were chosen; and were scored by Match/Mismatch of 1, −3, and Gap cost of Existence:5, Extension:2. Summarized result of GeNomad and TnCentral analysis were visualized using TBtools-II (v.2.326) [41]. Genomic Islands (GIs) of Vaw-5 were predicted by referencing the chromosome I of *Vibrio parahaemolyticus str.* RIMD 2210633 using the web version of IslandViewer 4 [42] with both the IslandPath-DIMOB [43] and SIGI-HMM [44] methods (accessed on 2 October 2025).

## 5. Conclusions

*V. parahaemolyticus* constitutes a principal factor in seafood-associated gastroenteritis on a global scale, correlated with occurrences stemming from uncooked or insufficiently cooked shellfish and tainted aquatic commodities [1]. The persistence of this pathogen in biofilm communities within seafood markets—as demonstrated by the Vaw-5 isolate—highlights an underappreciated reservoir that may contribute to the recurrence of foodborne infections and the dissemination of mobilizable resistance elements.

The supplementary genome of strain Vaw-5 has been modified by considerable lateral gene transfer, culminating in the establishment of distinctive genomic islands and transposable genetic elements. This investigation presents the most exhaustive genomic examination of a biofilm-adapted *V. parahaemolyticus* variant to this point in time. Our analysis of this environmental isolate and its clinical counterparts reveals a radically different adaptation strategy that prioritizes physiological processes such as efflux pumps, structural biofilm barriers, and a latent potential for gene mobilization over the acquisition of traditional antibiotic resistance genes. Despite genomic information indicating modifications associated with exopolysaccharide production, stress reactions, and phage interactions, numerous apprehensions remain unresolved regarding the phenotypic manifestation of these characteristics and their genuine ecological functions. Despite its limited ARG profile, Vaw-5′s potential role as a reservoir for resistance determinants in a One Health framework is underscored by the presence of mobilizable elements, such as transposons *Tn5501* and *Tn5393*, adjacent to resistance genes. To thoroughly grasp the processes underlying *V. parahaemolyticus* biofilm adaptability, forthcoming inquiries must integrate functional genomics, transcriptomics, and empirical validation with enhanced environmental monitoring. To effectively translate these genetic insights into control methods that limit the threats to public health presented by biofilm-associated pathogens in the food chain and their role in the antibiotic resistance dilemma, a multidisciplinary approach is required.

## Figures and Tables

**Table 1:** Multilocus Sequence Typing (MLST) profiles of the *Vibrio parahaemolyticus* strains. The table details the allele numbers for the seven standard housekeeping genes (*dnaE*, *gyrB*, *recA*, *dtdS*, *pntA*, *pyrC*, *tnaA*) and the resulting Sequence Type (ST). Strain Vaw-5 is assigned to ST-424, which it shares with an animal-associated isolate (GCA_021729965.1). N.A: Not Assigned; N.D: Not Detected.

	Scheme	ST	1	2	3	4	5	6	7
GCA_015172915.1_environmental	*V. parahaemolyticus*	N.A	*dnaE(330)*	*gyrB(268)*	*recA(202)*	*dtdS(227)*	*pntA(4)*	*pyrC(441)*	*tnaA(145)*
GCA_026650865.1_environmental	*V. parahaemolyticus*	N.A	*dnaE(44)*	*gyrB(106)*	*recA(393)*	*dtdS(126)*	*pntA(28)*	*pyrC(268)*	*tnaA(193)*
GCA_009883815.1_animals	*V. parahaemolyticus*	N.A	*dnaE(42)*	*gyrB(134)*	*recA(99)*	*dtdS(460)*	*pntA(26)*	*pyrC(41)*	*tnaA(51)*
GCA_030994225.1_animals	*Vibrio*	N.A	*gyrB(83)*	*pyrH(36)*	*recA(62)*	*atpA(60)*	***N.D***	***N.D***	***N.D***
GCA_008693625.1_clinical	*V. parahaemolyticus*	N.A	*dnaE(26)*	*gyrB(16)*	*recA(56)*	*dtdS(157)*	*pntA(4)*	*pyrC(32)*	*tnaA(51)*
GCA_001558495.2_clinical	*V. parahaemolyticus*	1	*dnaE(5)*	*gyrB(52)*	*recA(27)*	*dtdS(13)*	*pntA(17)*	*pyrC(25)*	*tnaA(10)*
GCA_030552955.1_clinical	*V. parahaemolyticus*	3	*dnaE(3)*	*gyrB(4)*	*recA(19)*	*dtdS(4)*	*pntA(29)*	*pyrC(4)*	*tnaA(22)*
GCA_028228685.1_clinical	*V. parahaemolyticus*	3	*dnaE(3)*	*gyrB(4)*	*recA(19)*	*dtdS(4)*	*pntA(29)*	*pyrC(4)*	*tnaA(22)*
GCA_002073775.2_clinical	*V. parahaemolyticus*	3	*dnaE(3)*	*gyrB(4)*	*recA(19)*	*dtdS(4)*	*pntA(29)*	*pyrC(4)*	*tnaA(22)*
GCA_000196095.1_clinical	*V. parahaemolyticus*	3	*dnaE(3)*	*gyrB(4)*	*recA(19)*	*dtdS(4)*	*pntA(29)*	*pyrC(4)*	*tnaA(22)*
GCA_004006515.1_animals	*V. parahaemolyticus*	3	*dnaE(3)*	*gyrB(4)*	*recA(19)*	*dtdS(4)*	*pntA(29)*	*pyrC(4)*	*tnaA(22)*
GCA_000430405.1_animals	*V. parahaemolyticus*	23	*dnaE(17)*	*gyrB(16)*	*recA(13)*	*dtdS(36)*	*pntA(15)*	*pyrC(31)*	*tnaA(26)*
GCA_001188185.2_clinical	*V. parahaemolyticus*	36	*dnaE(21)*	*gyrB(15)*	*recA(1)*	*dtdS(23)*	*pntA(23)*	*pyrC(21)*	*tnaA(16)*
GCA_030291875.1_clinical	*V. parahaemolyticus*	224	*dnaE(28)*	*gyrB(83)*	*recA(82)*	*dtdS(117)*	*pntA(18)*	*pyrC(69)*	*tnaA(79)*
GCA_001879585.1_processedfood	*V. parahaemolyticus*	233	*dnaE(109)*	*gyrB(136)*	*recA(114)*	*dtdS(121)*	*pntA(83)*	*pyrC(107)*	*tnaA(83)*
GCA_015779285.1_animals	*V. parahaemolyticus*	413	*dnaE(47)*	*gyrB(8)*	*recA(166)*	*dtdS(19)*	*pntA(28)*	*pyrC(46)*	*tnaA(121)*
GCA_009883835.1_animals	*V. parahaemolyticus*	415	*dnaE(42)*	*gyrB(134)*	*recA(99)*	*dtdS(79)*	*pntA(26)*	*pyrC(41)*	*tnaA(51)*
GCA_021729965.1_animals	*V. parahaemolyticus*	424	*dnaE(170)*	*gyrB(224)*	*recA(75)*	*dtdS(139)*	*pntA(117)*	*pyrC(18)*	*tnaA(124)*
Vaw-5_environmental	*V. parahaemolyticus*	424	*dnaE(170)*	*gyrB(224)*	*recA(75)*	*dtdS(139)*	*pntA(117)*	*pyrC(18)*	*tnaA(124)*
GCA_000568495.1_environmental	*V. parahaemolyticus*	471	*dnaE(175)*	*gyrB(22)*	*recA(168)*	*dtdS(201)*	*pntA(130)*	*pyrC(17)*	*tnaA(73)*
GCA_030345035.1_environmental	*V. parahaemolyticus*	624	*dnaE(3)*	*gyrB(4)*	*recA(73)*	*dtdS(13)*	*pntA(4)*	*pyrC(214)*	*tnaA(33)*
GCA_023205955.1_environmental	*V. parahaemolyticus*	722	*dnaE(26)*	*gyrB(16)*	*recA(234)*	*dtdS(7)*	*pntA(18)*	*pyrC(32)*	*tnaA(7)*
GCA_000430425.1_clinical	*V. parahaemolyticus*	799	*dnaE(28)*	*gyrB(4)*	*recA(82)*	*dtdS(88)*	*pntA(63)*	*pyrC(187)*	*tnaA(1)*
GCA_001244315.1_animals	*V. parahaemolyticus*	984	*dnaE(49)*	*gyrB(209)*	*recA(249)*	*dtdS(50)*	*pntA(112)*	*pyrC(37)*	*tnaA(23)*
GCA_033100075.1_environmental	*V. parahaemolyticus*	1160	*dnaE(153)*	*gyrB(191)*	*recA(70)*	*dtdS(19)*	*pntA(6)*	*pyrC(8)*	*tnaA(1)*
GCA_001433415.1_environmental	*V. parahaemolyticus*	1628	*dnaE(111)*	*gyrB(320)*	*recA(22)*	*dtdS(34)*	*pntA(20)*	*pyrC(21)*	*tnaA(24)*
GCA_001636035.1_animals	*V. parahaemolyticus*	1629	*dnaE(225)*	*gyrB(104)*	*recA(226)*	*dtdS(201)*	*pntA(50)*	*pyrC(250)*	*tnaA(17)*
GCA_001304775.1_enviromental	*V. parahaemolyticus*	1630	*dnaE(31)*	*gyrB(106)*	*recA(135)*	*dtdS(402)*	*pntA(37)*	*pyrC(212)*	*tnaA(54)*
GCA_014217295.1_animals	*V. parahaemolyticus*	1743	*dnaE(112)*	*gyrB(8)*	*recA(61)*	*dtdS(425)*	*pntA(26)*	*pyrC(8)*	*tnaA(57)*
GCA_023206515.1_environmental	*V. parahaemolyticus*	1750	*dnaE(103)*	*gyrB(490)*	*recA(31)*	*dtdS(169)*	*pntA(26)*	*pyrC(401)*	*tnaA(79)*
GCA_023205895.1_environmental	*V. parahaemolyticus*	1805	*dnaE(47)*	*gyrB(91)*	*recA(166)*	*dtdS(46)*	*pntA(79)*	*pyrC(45)*	*tnaA(26)*
GCA_001996365.2_animals	*V. parahaemolyticus*	1913	*dnaE(363)*	*gyrB(505)*	*recA(218)*	*dtdS(442)*	*pntA(30)*	*pyrC(303)*	*tnaA(26)*
GCA_001758605.1_clinical	*V. parahaemolyticus*	2015	*dnaE(67)*	*gyrB(522)*	*recA(31)*	*dtdS(70)*	*pntA(47)*	*pyrC(436)*	*tnaA(17)*

**Table 2:** Metadata and source information for the *Vibrio parahaemolyticus* strains used in the comparative genomic analysis. The dataset includes the novel environmental biofilm isolate Vaw-5 and 32 publicly available genomes representing diverse isolation sources (clinical, animal, environmental, processed food), geographical origins, and collection dates.

Sample	Type	Source	Country	Collection Date
GCA_000196095.1_clinical.fna	clinical	Human	Japan	1996
GCA_000430405.1_animals.fna	animals	Bivalves	USA	2007
GCA_000430425.1_clinical.fna	clinical	Human	USA	2006
GCA_000568495.1_environmental.fna	environmental	Sediment	Spain	2002
GCA_001188185.2_clinical.fna	clinical	Human	USA	1998
GCA_001244315.1_animals.fna	animals	Fish	South Korea	2014
GCA_001304775.1_environmental.fna	environmental	Surface	South Korea	2014
GCA_001433415.1_environmental.fna	environmental	Water	South Korea	2014
GCA_001558495.2_clinical.fna	clinical	Human	Japan	1951
GCA_001636035.1_animals.fna	animals	Fish	South Korea	2015
GCA_001758605.1_clinical.fna	clinical	Human	South Korea	2014
GCA_001879585.1_processedfood.fna	processed food	Crab	South Korea	Not available
GCA_001996365.2_animals.fna	animals	Shrimp	Malaysia	2016
GCA_002073775.2_clinical.fna	clinical	Human	India	1996
GCA_004006515.1_animals.fna	animals	Processed food	China	2012
GCA_008693625.1_clinical.fna	clinical	Human	USA	Not available
GCA_009883815.1_animals.fna	animals	Shrimp	China	2014
GCA_009883835.1_animals.fna	animals	Shrimp	China	2014
GCA_014217295.1_animals.fna	animals	Shrimp	China	2017
GCA_015172915.1_environmental.fna	environmental	Water	South Korea	2019
GCA_015779285.1_animals.fna	animals	Shrimp	China	2020
GCA_021729965.1_animals.fna	animals	Shrimp	China	2017
GCA_023205895.1_environmental.fna	environmental	Water	China	2019
GCA_023205955.1_environmental.fna	environmental	Water	China	2018
GCA_023206515.1_environmental.fna	environmental	Water	China	2018
GCA_026650865.1_environmental.fna	environmental	Water	China	2020
GCA_028228685.1_clinical.fna	clinical	Human	Thailand	2021
GCA_030291875.1_clinical.fna	clinical	Human	China	2015
GCA_030345035.1_environmental.fna	environmental	Water	South Korea	2022
GCA_030552955.1_clinical.fna	clinical	Human	China	2009
GCA_030994225.1_animals.fna	animals	Bivalves	Colombia	2021
GCA_033100075.1_environmental.fna	environmental	Water	China	2023
Vaw-5_environmental.fna	environmental	Biofilm	China	2023

## Data Availability

The datasets analyzed during the current study are publicly available in the NCBI repository (https://www.ncbi.nlm.nih.gov/sra/docs/) (accessed on 19 November 2024) for the following BioProject numbers: PRJNA360; PRJNA203445; PRJNA203445; PRJNA229758; PRJNA231221; PRJNA266097; PRJNA261558; PRJNA259940; PRJNA231221; PRJNA280138; PRJNA284329; PRJNA301198; PRJNA355061; PRJNA231221; PRJNA487159; PRJNA231221; PRJNA503785; PRJNA503785; PRJNA655788; PRJNA662942; PRJNA681469; PRJNA503785; PRJNA633360; PRJNA633360; PRJNA633360; PRJNA691968; PRJNA866285; PRJNA982304; PRJNA983515; PRJNA997237; PRJNA852548; PRJNA1028838; PRJNA1112528.

## Supplementary Materials

The following supporting information can be downloaded at: https://www.mdpi.com/article/10.3390/antibiotics14101005/s1, Figure S1: Distance trees of CLUSTER0239—*yeiP* elongation factor G across all 33 *Vibrio parahaemolyticus* strains; Figure S2: Distance trees of CLUSTER0888—DNA repair protein *RecO* across all 33 *Vibrio parahaemolyticus* strains; Figure S3: Distance trees of CLUSTER0600—virulence factor *BrkB* family protein across all 33 *Vibrio parahaemolyticus* strains; Figure S4: Distance trees of CLUSTER3016—*OmpH* family outer membrane protein across all 33 *Vibrio parahaemolyticus* strains; Table S1. Summary of Genomic Islands (GIs) in the *Vibrio parahaemolyticus* Vaw-5 genome. The table provides the start and end positions and length for each GI. Detailed annotations for all genes contained within each GI are listed, including gene ID, locus tag, genomic coordinates, strand, and predicted product/function. Gene names from external databases are included where available.

## Abbreviations

The following abbreviations are used in this manuscript:

ABRicate	A tool for detecting antimicrobial resistance and virulence genes
ARGs	Antibiotic Resistance Genes
AHPND	Acute Hepatopancreatic Necrosis Disease
CRISPR-Cas	Clustered Regularly Interspaced Short Palindromic Repeats and CRISPR-associated proteins
DNA	Deoxyribonucleic Acid
GCA	GenBank Assembly Accession prefix
HGT	Horizontal Gene Transfer
IS	Insertion Sequence
MGEs	Mobile Genetic Elements
MLST	Multilocus Sequence Typing
NCBI	National Center for Biotechnology Information
NGS	Next-Generation Sequencing
OmpH	Outer Membrane Protein H
PCR	Polymerase Chain Reaction
Prokka	A software tool for prokaryotic genome annotation
qnrVC	Quinolone resistance gene variant
Refseq	Reference Sequence database
ResFinder	A database for antibiotic resistance genes
Roary	A pangenome analysis tool
ST	Sequence Type
TBtools	A bioinformatics software toolkit
*tdh/trh*	Thermostable Direct Hemolysin/TDH-related hemolysin genes
*Tn5501*, *Tn5393*	Transposon identifiers
TORMES	A pipeline for bacterial genome analysis
VFDB	Virulence Factor Database
Vaw-5	*Vibrio parahaemolyticus* strain name
