# Supplementary material for: Genomic Insights into an Environmental Vibrio parahaemolyticus Biofilm Isolate: Deciphering Alternative Resistance Mechanisms and Mobilizable Genetic Elements
Source: Antibiotics (Basel). 2025 Oct 10;14(10):1005. doi: 10.3390/antibiotics14101005 (PMC12561420; doi:10.3390/antibiotics14101005)

YeiP elongation factor G - housekeeping gene

Distance tree (Muscle+DnaDist+FastME) of the different genes of the cluster (across the different strains).

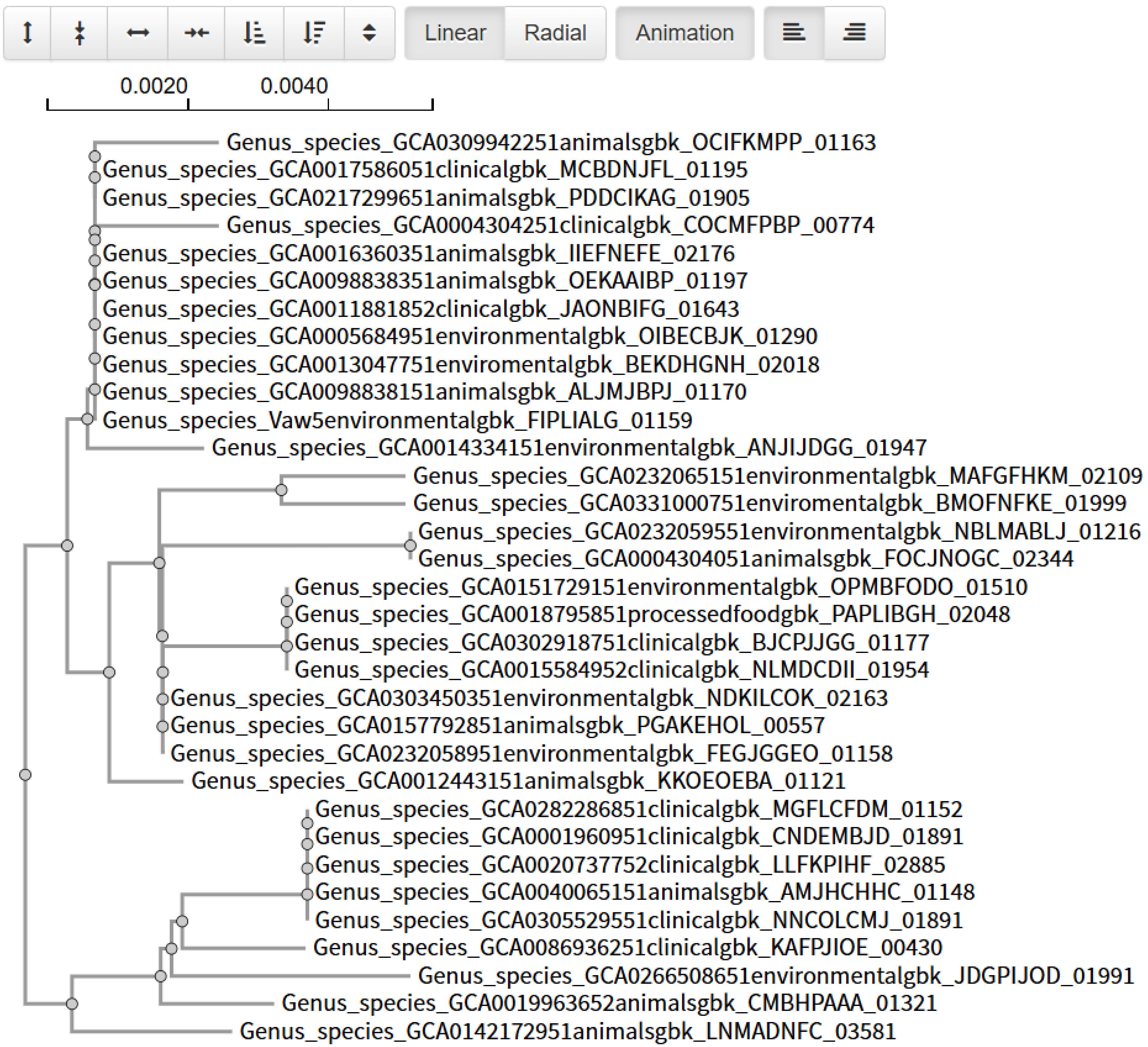

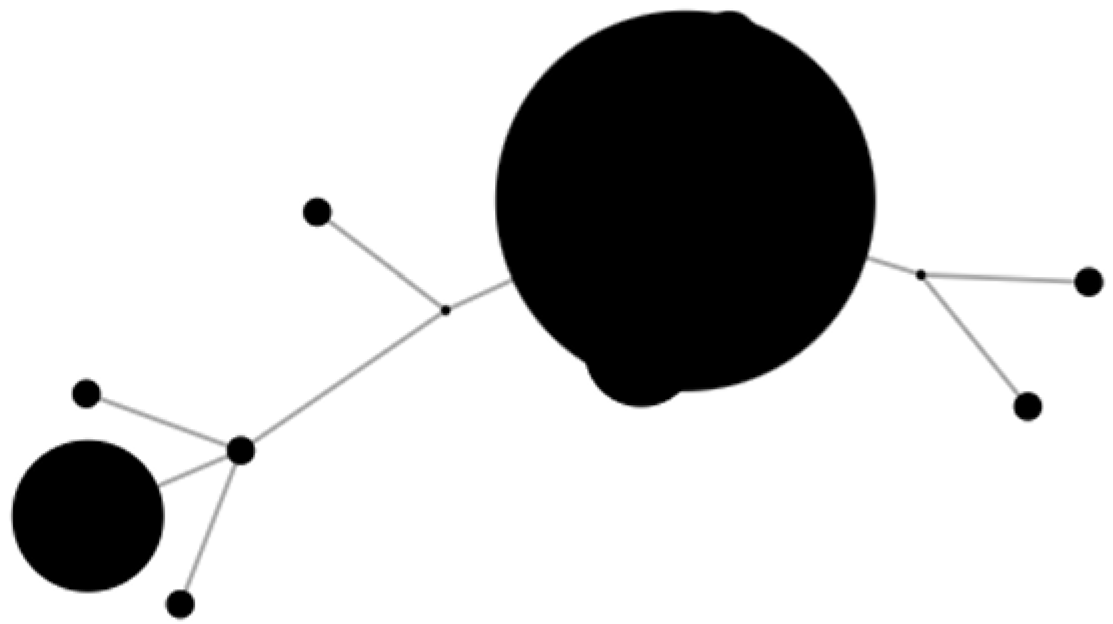

Supplement: Supplementary file 1 [file antibiotics-14-01005-s001.zip › antibiotics-3859768-supplementary/antibiotics-3859768-Supplementary Material/Sup. Fig1.yeip.pdf]
